# Decoupling anion-ordering and spin-Peierls transitions in a strongly one-dimensional organic conductor with a chessboard structure, (*o*-Me_2_TTF)_2_NO_3_

**DOI:** 10.1107/S2052252518004967

**Published:** 2018-04-27

**Authors:** Olivier Jeannin, Eric W. Reinheimer, Pascale Foury-Leylekian, Jean-Paul Pouget, Pascale Auban-Senzier, Elzbieta Trzop, Eric Collet, Marc Fourmigué

**Affiliations:** a Université de Rennes, CNRS, ISCR (Institut des Sciences Chimiques de Rennes), UMR 6226, Rennes F-35000, France; b Laboratoire de Physique des Solides, Université Paris-Saclay, CNRS, Université Paris-Sud, UMR 8502, Orsay 91405, France; c Université de Rennes, CNRS, IPR (Institut de Physique de Rennes), UMR 6251, Rennes F-35000, France

**Keywords:** anion-ordering transition, spin-Peierls transition, organic conductors, molecular crystals, phase transitions

## Abstract

A mixed-valence conducting cation radical salt of the unsymmetrically substituted *o*-Me_2_TTF donor molecule (TTF is tetrathiafulvalene) was obtained upon electrocrystallization in the presence of the non-centrosymmetric NO_3_^−^ anion. It crystallizes at room temperature in the monoclinic *P*2_1_/*c* space group, with the anion disordered on an inversion centre. The donor molecules are stacked along the *a* axis. A 90° rotation of the longest molecular axis of *o*-Me_2_TTF generates a chessboard-like structure, preventing lateral S⋯S contacts between stacks and providing a strongly one-dimensional electronic system, as confirmed by overlap interaction energies and band structure calculations. A strong dimerization within the stacks explains the semi­conducting behaviour of the salt, with σ_room temp_ = 3–5 S cm^−1^ and *E*_activated_ = 0.12–0.14 eV. An X-ray diffuse scattering survey of reciprocal space, combined with full structure resolutions at low temperatures (250, 85 and 20 K), evidenced the succession of two structural transitions: a ferroelastic one with an anion-ordering (AO) process and the establishment of a (0, ½, ½) superstructure below 124 (±3) K, also visible *via* resistivity thermal dependence, followed by a stack tetramerization with the establishment of a (½, ½, ½) superstructure below 90 (±5) K. The latter ground state is driven by a spin-Peierls (SP) instability, as demonstrated by the temperature dependence of the magnetic susceptibility. Surprisingly, these two kinds of instability appear to be fully decoupled here, at variance with other tetra­methyl­tetra­thia­fulvalene (TMTTF) or tetramethyl­tetra­selena­fulvalene (TMTSF) salts with such non-centrosymmetric counter-ions.

## Abstract 2

Anion-ordering (AO) and spin-Peierls (SP) structural/electronic transitions are fully decoupled in the molecular conductor (*o*-Me_2_TTF)_2_NO_3_.

## 1. Introduction   

**Figure 1:**
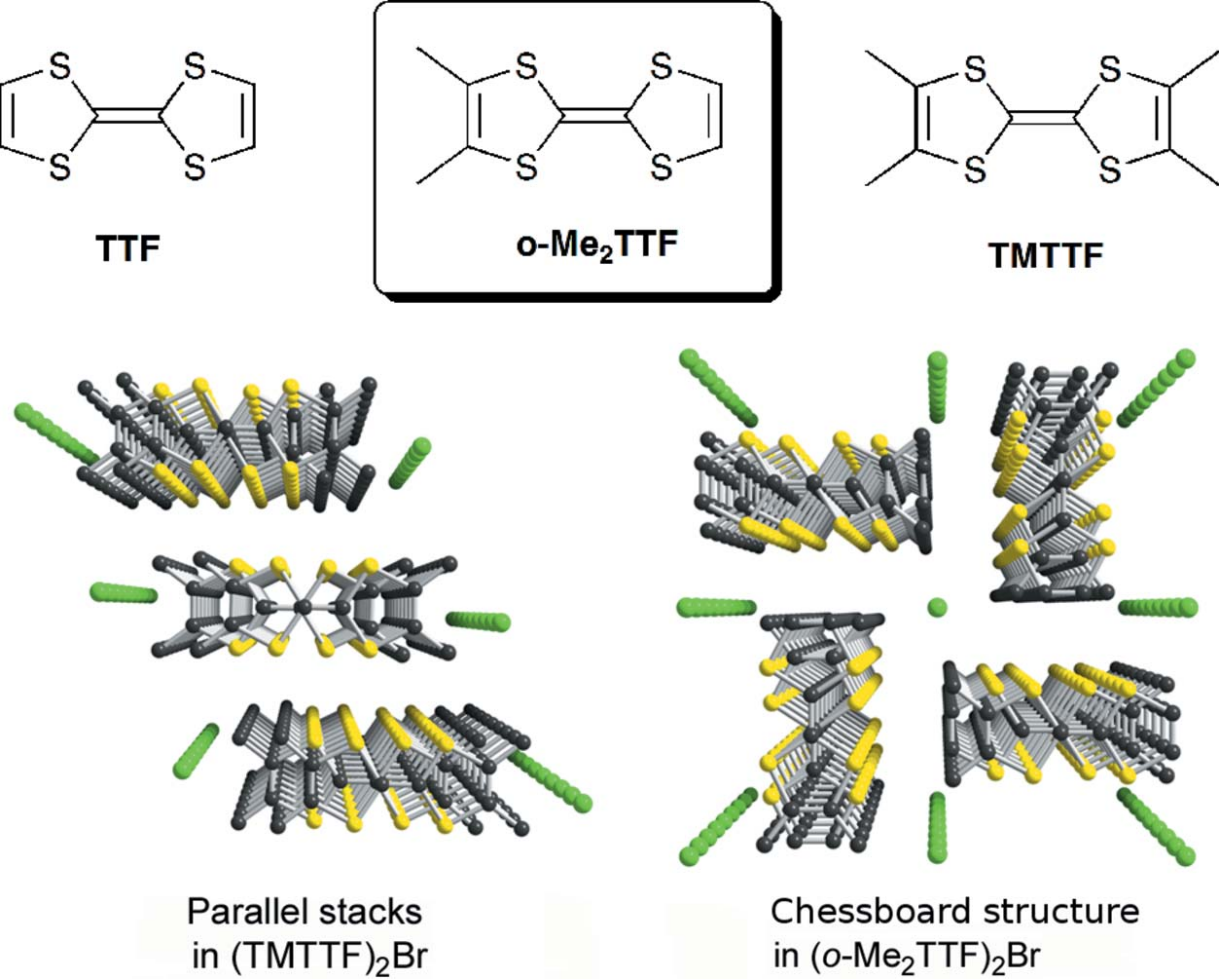
Perspective views along the stacking axis of two different salts, (TMTTF)_2_Br with parallel stacks, and (*o*-Me_2_TTF)_2_Br with a chessboard structure.

Organic conductors based on tetra­thia­fulvalene derivatives (Batail, 2004 ▸) most often adopt a so-called 2:1 stoichiometry, with two donor molecules for one counter-ion, as found in the extensive series of Fabre salts, formulated as (TMTTF)_2_ *X* (TMTTF is tetra­methyl­tetra­thia­fulvalene; *X* = Br^−^, ClO_4_^−^, NO_3_^−^, PF_6_^−^, AsF_6_^−^, …). In these salts, the partially oxidized TMTTF derivatives stack on top of each other, while the overlap interaction between the highest occupied molecular orbitals (HOMOs) of TMTTF leads to the formation of a one-dimensional conduction band, formally three-quarters filled with this 2:1 stoichiometry. One important parameter is the degree of dimerization within these chains, as dimerization opens a gap at mid filling and allows the upper band to be half-filled, with important consequences for the associated electronic properties (Jérome, 2004 ▸) and with a very rich sequence of competing ground states. As stated by Giamarchi (2004 ▸), most of the physics of these quasi-one-dimensional systems stems from the coupling between the chains. Indeed, the dominant one-dimensional character at high temperatures (and/or low pressure) evolves at lower temperatures (and/or higher pressures) towards a three-dimensional regime [with the appearance of ordered states such as charge ordering, spin-Peierls (SP), antiferromagnetism and superconductivity] through an intermediate two-dimensional electronic regime well noted for the isostructural Bechgaard salts based on the TMTSF donor (TMTSF is tetra­methyl­tetra­selena­fulvalene). Therefore, it is highly desirable to have at hand systems where the inter­actions between stacks are not only very weak but also isotropic (in the plane perpendicular to the stacking axis), in order to favour a direct one- to three-dimensional crossover and to allow a proper evaluation of the actual role of this two-dimensional electronic coupling. In that respect, we have recently investigated a series of conducting halide salts derived from an unsymmetrically substituted TTF, *o*-Me_2_TTF, formulated as (*o*-Me_2_TTF)_2_ *X* (*X* = Cl, Br, I; Fourmigué *et al.*, 2008 ▸), and which crystallize in the 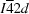 space group. In these salts, the donor molecules form strictly regular, *i.e.* non-dimerized, stacks. A 90° rotation of the longest molecular axis of *o*-Me_2_TTF generates a chessboard-like structure, a very original motif which differs strongly from that of TMTTF salts where the donor stacks organize parallel to each other (Fig. 1 ▸). As a consequence of the chessboard structure of (*o*-Me_2_TTF)_2_ *X* salts, the inter-stack interactions are very weak, as revealed by their high conductivity anisotropy (Foury-Leylekian *et al.*, 2011 ▸).

Considering the strong interest raised by the halide salts of the *o*-Me_2_TTF molecule (Fourmigué *et al.*, 2008 ▸; Foury-Leylekian *et al.*, 2011 ▸), we decided to investigate in more detail its salts with other anions. Before our work on halide salts of *o*-Me_2_TTF, only a 1:1 insulating salt with ReO_4_^−^ had been structurally characterized (Mhanni *et al.*, 1993 ▸), while several conducting 2:1 salts were reported as early as 1983, without X-ray crystal structure resolution, with the BF_4_^−^, ClO_4_^−^ and SCN^−^ anions (Abderraba *et al.*, 1983 ▸). However, all our previous attempts with, for example, BF_4_^−^ (Reinheimer, Zhao, & Dunbar, 2008 ▸), I_3_^−^ (Reinheimer *et al.*, 2009 ▸), Re_2_Cl_8_^2−^ (Reinheimer, Galán-Mascarós *et al.*, 2008 ▸) or W_6_O_19_^2−^ (Reinheimer *et al.*, 2013 ▸) led to insulating salts of the fully oxidized *o*-Me_2_TTF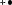 cations, associated into diamagnetic dicationic dyads (*o*-Me_2_TTF)_2_^2+^. On the other hand, the non-centrosymmetric NO_3_^−^ nitrate anion affords a conducting 2:1 salt (*o*-Me_2_TTF)_2_(NO_3_), furthermore characterized by the chessboard arrangement of strongly one-dimensional organic stacks (see Fig. 1 ▸) but also with a disorder of the NO_3_^−^ anion. In this paper we show, by combining electrical, magnetic and structural measurements, that (*o*-Me_2_TTF)_2_(NO_3_) exhibits a very original phase diagram among the various series of one-dimensional conductors, with an anion-ordering (AO) process in two steps and a spin-Peierls (SP) transition towards a spin singlet non-magnetic ground state.

## 2. Experimental   

### 2.1. Synthesis and crystal growth   

*o*-Me_2_TTF was prepared as previously described through a selective cross-coupling reaction (Gerson *et al.*, 1996 ▸) involving a modified Horner–Edmonds reaction, which affords the unsymmetrically substituted molecule without any trace of the symmetric TTF or Me_4_TTF products, at variance with the original synthesis based on the statistical cross-coupling reaction of the di­thiol­ium and 4,5-dimethyldithiolium cations in the presence of NEt_3_ (Wudl *et al.*, 1977 ▸).

### 2.2. Electrocrystallization   

*o*-Me_2_TTF (10 mg) was oxidized on a Pt electrode (1 cm long, 0.5 cm in diameter) in an electrolyte solution (0.1 *M*), prepared by dissolving [*n*-Bu_4_N][NO_3_] (0.3403, 0.100 mmol) in freshly distilled CH_3_CN (10 ml), at a constant current of 0.5 µA at a temperature of 275 K. After one week, black needle-like crystals of the 2:1 (*o*-Me_2_TTF)_2_NO_3_ salt were harvested from the anodic compartment of the electrochemical cell and washed with small amounts of EtOH.

### 2.3. X-ray diffraction studies   

X-ray diffuse scattering investigation was performed using a homemade three-circle diffractometer (normal beam geometry with a lifting linear Ar–CH_4_ gas detector) equipped with a three-stage closed-circuit He gas cryocooler operating from room temperature down to 1.8 K. The rotation of the sample was provided by a magnetic coupling system connected to the cryocooler head. The experimental setup was mounted on a rotating-anode X-ray generator operating at 50 kV and 50 mA and providing Cu *K*α (λ = 1.542 Å) radiation after Confocal Max-Flux optics (beam size ≃ 350 µm). Before the quantitative measurements, a preliminary photographic investigation using an image plate instead of the linear detector was systematically performed in order to detect easily any additional diffuse scattering or satellite reflections associated with any structural modification.

The crystallographic data for (*o*-Me_2_TTF)_2_NO_3_ at 20 K were collected with an Oxford Diffraction Xcalibur3 diffractometer fitted with a two-dimensional Sapphire3 CCD detector using sealed monochromatic Mo *K*α radiation source (λ = 0.71073 Å). The Xcalibur3 diffractometer was fitted with a helium-flow Helijet Oxford Diffraction cryostat. The crystallographic data for (*o*-Me_2_TTF)_2_NO_3_ at 85 and 250 K were collected with a Rigaku Oxford Diffraction SuperNova diffractometer fitted with an Eos-S2 detector using micro-focused monochromatic Cu *K*α radiation (λ = 1.54184 Å). The SuperNova diffractometer was fitted with a nitro­gen-flow Oxford Cryosystems 800Plus series device.

At 20, 85 and 250 K, data collection, cell-constant determination and data reduction were performed using the *CrysAlisPro* software (Rigaku Oxford Diffraction, 2015 ▸). All structure models were solved by dual-space direct methods (*SHELXT*; Sheldrick, 2015 ▸) and developed by full least-squares refinement on *F* ^2^ (*SHELXL*; Sheldrick, 2008 ▸, 2015 ▸) using *OLEX2* interfaces (Dolomanov *et al.*, 2009 ▸). Crystallographic figures were prepared using *DIAMOND* (Brandenburg, 2006 ▸). Anisotropic displacement parameters were used for all non-hydrogen atoms. Hydrogen atoms were added at the calculated positions and refined using a riding model. Experimental details and structure determination parameters are given in Table 1 ▸.

Where applicable, DFIX and DANG (restraints on the distances and angles to a target value with an estimated standard deviation) and SIMU and ISOR (restraints on the *U_ij_* components to be close to those of neighbouring atoms or close to isotropic behaviour) were used for modelling the geometry and anisotropic displacement parameters, respectively, of disordered NO_3_^−^ ions. Only in the case of the 20 K data was the ISOR restraint applied to the anisotropic displacement parameters for some carbon sites of *o*-Me_2_TTF molecules. It is of importance that, at 250 K, the NO_3_^−^ ion is disordered on an inversion centre. In the case of the 20 K data, the (*o*-Me_2_TTF)_2_NO_3_ crystal structure contains a two-component twin model with a ratio of 0.66:0.34; the two components are related by the twin matrix (−1 0 0, 0 0 −1, 0 −1 0). On the other hand, the crystal collected at 85 K was not twinned, despite having undergone a phase transition. Alerts A and B in the CIF arise because of the disorder or possible disorder of the NO_3_^−^ anions.

CCDC deposition numbers 1562992–1562994 for the crystals measured at 20 K, 85 K and 250 K, respectively, contain the supplementary crystallographic data for this paper. The data can be obtained free of charge from the Cambridge Crystallographic Data Centre (http://www.ccdc.cam.ac.uk/structures).

### 2.4. Resistivity measurements   

**Figure 2:**
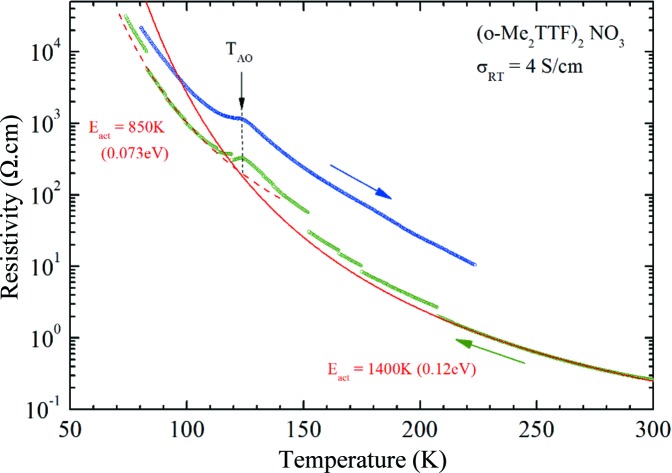
Temperature dependence of the longitudinal resistivity for (*o*-Me_2_TTF)_2_NO_3_. The red solid (dashed) curve is the fit to the high-temperature (low-temperature) data with ρ = ρ_0_ exp(*E*_act_/*T*). The higher resistivity observed in the warming cycle is attributed to cracks affecting the crystal during the initial cooling process.

To measure the longitudinal resistivity, gold pads were evaporated onto the surface of the single crystals in order to improve the quality of the contacts. The temperature dependence of the resistivity was then measured on a Quantum Design physical properties measurement system (PPMS) at a cooling or warming rate of 0.25 K min^−1^. The resistance was measured at four points with an applied current *I*_dc_ = 0.1 µA. When the measured resistance exceeded 100 kΩ, which occurred between 125 and 150 K depending on the sample, this current was lowered continuously in order to keep the voltage below 10 mV. Despite the slow cooling rate, some micro-cracks in the crystal induce jumps on the cooling curve, which explains the shift in the warming curve to a higher value of resistance observed in Fig. 2 ▸. Other single crystals were measured in a cryocooler equipment with a low-frequency lock-in detection (*I*_ac_ = 1 µA) for measured resistances below 100 kΩ and dc measurement for higher resistances (*I*_dc_ = 0.1–1 µA). The thermal dependence of their resistivity is qualitatively the same, despite a faster cooling rate (0.5 to 1 K min^−1^).

### 2.5. Theoretical calculations   

The tight-binding β_HOMO-HOMO_ interaction energy calculations were based upon the effective one-electron Hamiltonian of the extended Hückel method (Whangbo & Hoffmann, 1978 ▸), as implemented in the *Caesar 1.0* chain of programs (Ren *et al.*, 1998 ▸). The off-diagonal matrix elements of the Hamiltonian were calculated according to the modified Wolfsberg–Helmholz formula (Ammeter *et al.*, 1978 ▸). All valence electrons were explicitly taken into account in the calculations and the basis set consisted of double-ζ Slater-type orbitals for all atoms except H (simple-ζ Slater-type orbital) using the Roothaan–Hartree–Fock wavefunctions of Clementi & Roetti (1974 ▸).

## 3. Results   

### 3.1. Structural organization and electronic interactions   

**Figure 3:**
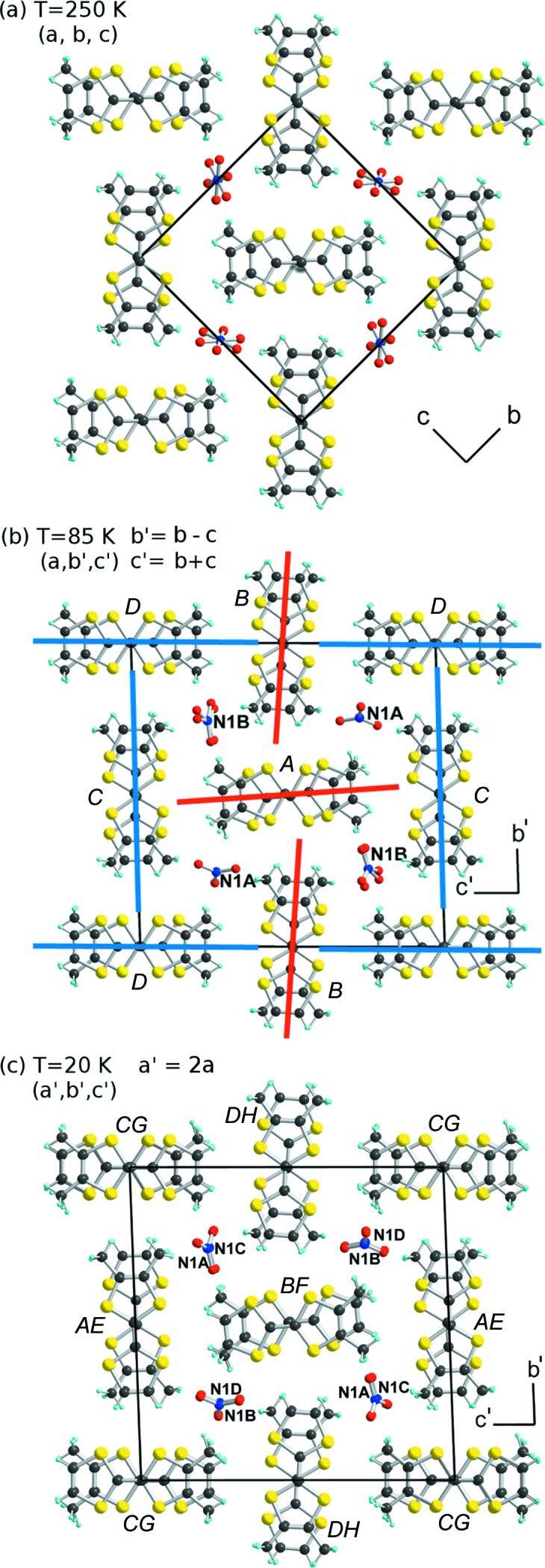
Projection views along **a** (or **a**′) of the unit cell of (*o*-Me_2_TTF)_2_NO_3_ at different temperatures. (*a*) *T* = 250 K. The NO_3_^−^ anions are disordered between two centrosymmetric positions. (*b*) *T* = 85 K in the *q*_1_ = (0, ½, ½) superstructure. Only the majority orientation (93.5%) of the nitrate anion on atom N1*A* is shown for clarity. The red and blue lines parallel to the molecular long axes illustrate the rotations of the stacks taking place in this intermediate *q*_1_ = (0, ½, ½) structure to accommodate the two different NO_3_^−^ orientations. (*c*) *T* = 20 K in the *q*_1_ + *q*_2_ = (½, ½, ½) superstructure.

The electrocrystallization of *o*-Me_2_TTF in the presence of [*n*-Bu_4_N^+^][NO_3_^−^] as electrolyte afforded a 2:1 salt formulated as (*o*-Me_2_TTF)_2_NO_3_. It crystallizes at 250 K (and above) in the monoclinic system, space group *P*2_1_/*c*, with the *o*-Me_2_TTF in a general position in the unit cell, while the NO_3_^−^ anion is disordered on an inversion centre. A projection view of the unit cell of (*o*-Me_2_TTF)_2_NO_3_ (Fig. 3 ▸ *a*) illustrates the general solid-state organization, with the organic stacks running along **a** and a 90° rotation of the long molecular axis in the nearest neighbouring stacks, affording a chessboard-like motif reminiscent of that mentioned above for the halide salts of *o*-Me_2_TTF (see Fig. 1 ▸).

**Figure 4:**
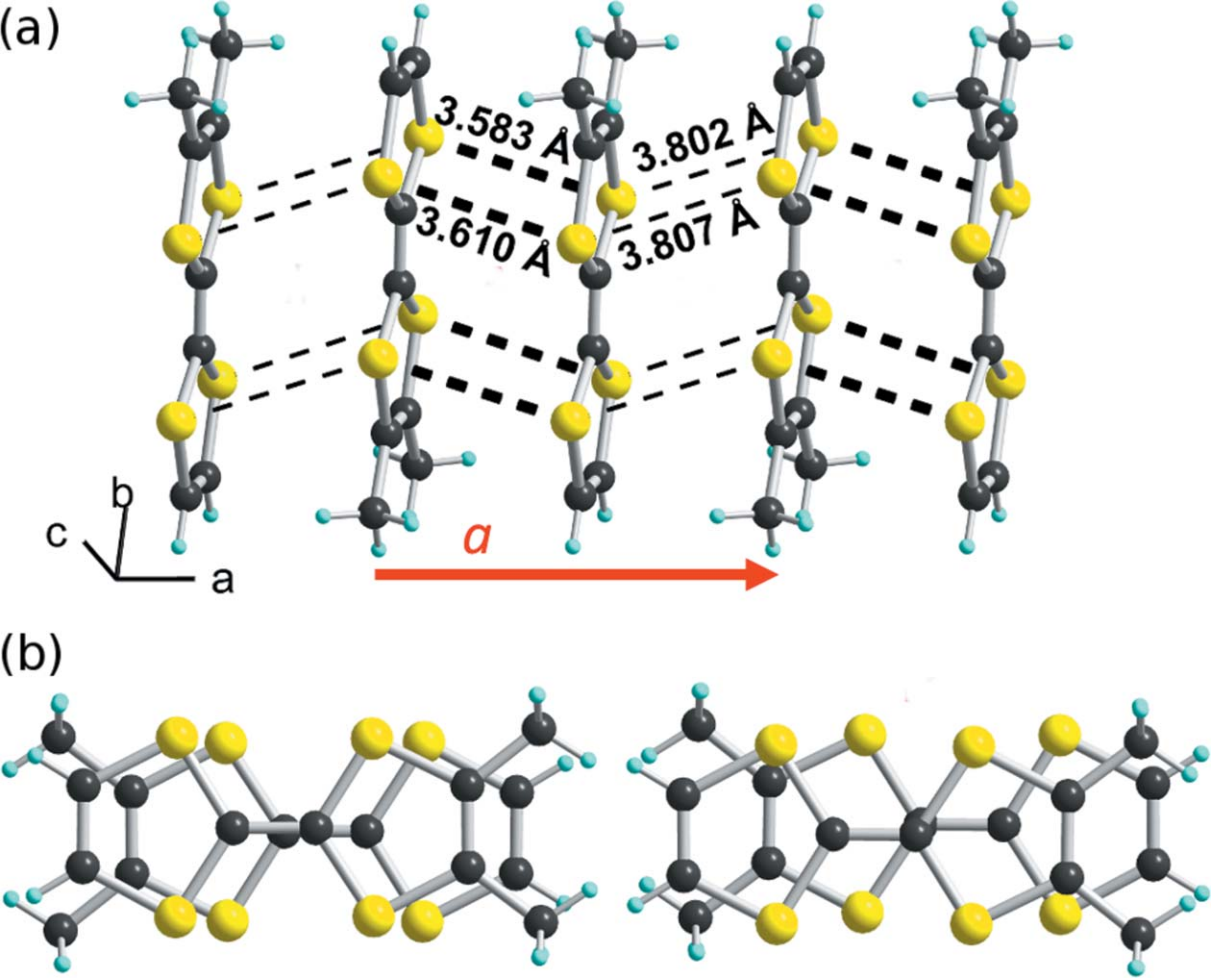
(*a*) A side view of one chain in (*o*-Me_2_TTF)_2_NO_3_ (stacking axis **a**) with intermolecular S⋯S contacts shown as black dotted lines. (*b*) The two different overlap patterns within one chain: (left) eclipsed and (right) bond-over-ring.

However, at variance with these halide salts with a uniform chain structure (Fig. 1 ▸ *b*), a side view of one stack (Fig. 4 ▸ *a*) shows that the organic columns are now strongly dimerized. In addition, an alternation of two types of overlap pattern is observed (Fig. 4 ▸ *b*): a strong overlap associated with an almost eclipsed conformation and short S⋯S distances, and a weaker overlap with a bond-over-ring overlap and long S⋯S (>3.8 Å) intermolecular distances (Fig. 4 ▸ *b*). This analysis is confirmed by the calculation of the β_HOMO-HOMO_ overlap interaction energies (Table 2 ▸), with a β_intra_ value associated with the eclipsed conformation and a weaker β_inter_ one associated with the bond-over-ring overlap. The degree of dimerization, defined as 2(β_intra_ − β_inter_)/(β_intra_ + β_inter_), is much larger here (0.6) than in the prototypical TMTTF salts (Liautard *et al.*, 1982*a* ▸; Galigné *et al.*, 1979 ▸). Indeed, it amounts to 0.38 in (TMTTF)_2_PF_6_ and only 0.11 in (TMTTF)_2_Br (Pouget & Ravy, 1996 ▸). The interactions between stacks are much weaker as the strongest one does not exceed 0.01 eV, which confirms the strong one-dimensional character of these salts.

### 3.2. Electrical conductivity   

**Figure 5:**
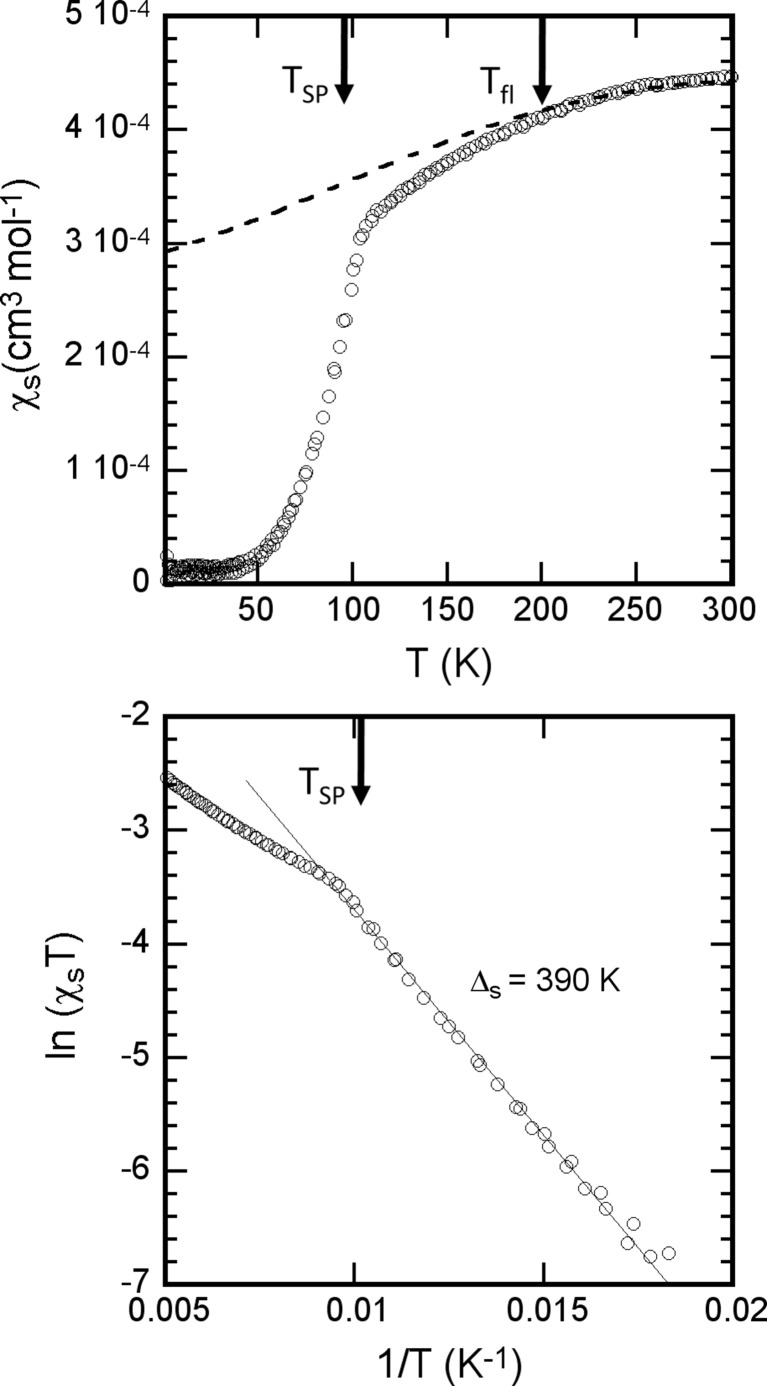
(*a*) Temperature dependence of the spin susceptibility, χ_s_, in (*o*-Me_2_TTF)_2_NO_3_. A Curie tail encompassing 0.65% *S* = ½ magnetic defaults has been subtracted. The dotted line is an HT fit to the uniform spin ½ *AF* Heisenberg chain. (*b*) Plot of ln(χ_s_ *T*) *versus* 1/*T*, allowing the determination of the spin activation energy Δ_σ_ ≃ 390 K in the spin-Peierls ground state.

Resistivity measurements performed on single crystals show (Fig. 2 ▸) a room-temperature conductivity of 3–5 S cm^−1^ and an activated behaviour upon cooling with ρ = ρ_0_exp(*E*_act_/*T*) and *E*_act_ = 0.12–0.14 eV determined above 150 K. The kink in the thermal dependence observed around 125 K is found in different investigated crystals, albeit to a varying extent, and is attributed to anion ordering. The maximum of the kink (called *T*_AO_ hereinafter) is observed at 122 (Fig. 5 ▸), 125 and 127 K for three different measured crystals. Below the kink, the activation energy decreases slightly (compare in Fig. 2 ▸ the activated thermal dependencies fitted above and below the kink). This activated conductivity with a large *E*_act_ means that, in spite of a three-quarters band filling, the electrons are localized with a gap of charge Δ_ρ_ ≃ 2*E*_act_. This electron localization is the combined result of the strong one-dimensional character of the band structure and the importance of electron–electron correlation in TTF-based systems enhanced by the stack dimerization (Giamarchi, 2004 ▸; Pouget, 2012*a* ▸, 2015 ▸). This behaviour should be contrasted with that of the (*o*-Me_2_TTF)_2_ *X* (*X* = Cl, Br, I) salts with a uniform stack, which exhibit a high-temperature metallic conductivity (Foury-Leylekian *et al.*, 2011 ▸).

### 3.3. Spin susceptibility   

The magnetic susceptibility was determined on a 15 mg sample of polycrystalline material. Fig. 5 ▸(*a*) reports the temperature dependence of its spin component, χ_σ_. Based on the crystal structure and semiconducting behaviour of this salt (see above), one expects over the whole temperature range a one-dimensional localized regime where each localized hole bears a spin ½. Thus, the thermal dependence of the spin susceptibility can be tentatively fitted in the high-temperature (HT) regime (*T* > 200 K) with a uniform spin chain Bonner–Fisher model which is associated with a *J*/*k* value of −520 (50) K (with the Hamiltonian 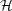 = 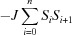) (Kahn, 1993 ▸). We note that, below 200 K, the susceptibility decays progressively from this uniform chain behaviour and then exhibits a sharp drop to a non-magnetic ground state at around 96 (±3) K. In the non-magnetic ground state (of the spin-Peierls type), the magnetic susceptibility is activated with a spin gap of Δ_σ_ ≃ 390 K (Fig. 5 ▸ *b*).

This abrupt magnetic transition is reminiscent of the phase transitions observed in the TMTTF salts with non-centrosymmetric anions (Coulon *et al.*, 1982 ▸, 2015 ▸), whether tetrahedral (ReO_4_^−^, ClO_4_^−^, BF_4_^−^), triangular (NO_3_^−^) or asymmetric linear (SCN^−^) anions. In all these salts, the location of the anions in crystal cavities delineated by the methyl groups provides enough softness and flexibility to accommodate disordering of the anions on inversion centres (Liautard *et al.*, 1982*b* ▸, 1983 ▸; Kistenmacher, 1984 ▸). Lowering the temperature can favour an anion-ordering (AO) transition with eventual cell parameter doubling in one, two or three directions (Pouget & Ravy, 1996 ▸). Recent examples also involve salts of unsymmetrical TTF derivatives such as DMEDO-TTF (3,4-di­methyl-3′,4′-ethyl­ene­dioxo­tetra­methyl­tetra­thia­fulvalene) with tetrahedral anions, ClO_4_^−^ and BF_4_^−^ (Kumeta *et al.*, 2016 ▸). They exhibit a first-order metal–insulator transition with anion ordering along the stacking axis. Besides, in charge-localized TMTTF salts with centrosymmetric anions such as PF_6_^−^ or AsF_6_^−^, an abrupt magnetic transition towards a spin-Peierls (SP) ground state also occurs (Pouget *et al.*, 2006 ▸). A thorough structural analysis is therefore required to differentiate between these two possibilities in (*o*-Me_2_TTF)_2_NO_3_.

### 3.4. Structural phase transitions   

**Figure 6:**
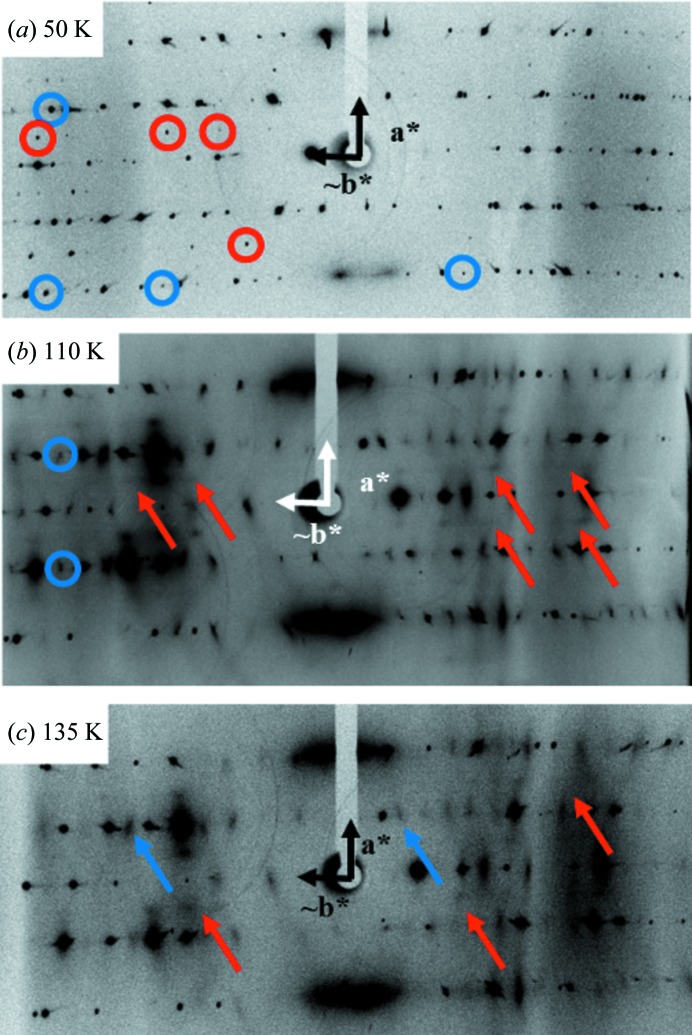
Typical X-ray patterns taken in the three phases. (*a*) At 50 K, for this X-ray pattern the λ/2 contribution has been suppressed. The red circles indicate the (½ 0 0) satellite reflections and the blue circles indicate the (0 ½ ½) satellite reflections. (*b*) At 110 K, the red arrows indicate the diffuse lines associated with the regime of quasi-one-dimensional fluctuations of the SP transition and the blue circles indicate the (0 ½ ½) satellite reflections. (*c*) At 135 K, the red arrows indicate the diffuse lines associated with the regime of quasi-one-dimensional fluctuations of the SP transition and the blue arrows show the isotropic regime of fluctuation associated with the anion ordering.

In order to obtain information on the evolution of the crystal structure at the various anomalies observed in the thermal dependence of the conductivity (Fig. 2 ▸) and of the magnetic susceptibility (Fig. 5 ▸), an X-ray diffuse scattering survey of reciprocal space was performed using the photographic method. Below 90 K, two sets of superstructure diffraction reflections are observed at the reduced wave­vectors *q*_1_ = (0, ½, ½) and *q*_2_ = (½, 0, 0) (Fig. 6 ▸ *a*). The components of these superstructure reflections are given as fractions of the high-temperature reciprocal wavevectors. The intensity of the mean *q*_1_ superstructure reflections is ∼10% of the average intensity of the Bragg reflections of the high-temperature lattice. Concerning the *q*_2_ superstructure reflections, their mean intensity is ∼15% that of the average Bragg intensity. The *q*_2_ superstructures, which correspond to a unit-cell doubling along the stack axis, vanish between 90 K (X-ray diffuse scattering investigation) and 85 K (diffractometric study – see below). So combining these measurements with the drop in spin susceptibility at 96 (±3) K (see section 3.3), one obtains a critical temperature for the SP transition of *T*_SP_ = 90 (5) K. In the intermediate temperature range between *T*_SP_ and 122 K (Fig. 6 ▸ *b*), only *q*_1_ superstructure reflections persist, while the *q*_2_ superstructure reflections have been transformed into an anisotropic quasi-one-dimensional diffuse scattering appearing as diffuse lines at the reduced *q*_2_ = (½, 0, 0) reciprocal position on the X-ray patterns. The *q*_1_ superstructure reflections, which correspond to a unit-cell doubling in directions perpendicular to the stack axis, vanish at 122 (2) K, which is the anion-ordering temperature *T*_AO_. At this structural transition there is a corresponding kink at 122–127 K in the thermal dependence of the resistivity (see Fig. 2 ▸). Above *T*_AO_, pre-transitional structural fluctuations are detected on the X-ray patterns (Fig. 6 ▸ *c*). Broad *q*_1_ pre-transitional diffuse spots remain observable for more than 15 K above *T*_AO_, while the *q*_2_ = (½, 0, 0) diffuse lines, precursors of the *T*_SP_ transition, are observed over a much larger temperature range until ∼200 K (*T*_fl_).

### 3.5. Superstructure structural refinement   

The X-ray diffuse scattering data were complemented by full single-crystal X-ray data collection and structural refinement performed at 85 and 20 K.

#### 3.5.1. The *q*_1_ = (0, ½, ½) superstructure refinement   

X-ray data collection was performed at 85 K, slightly below *T*_SP_, because the *q*_1_ = (0, ½, ½) superstructure intensity was the strongest and the additional *q*_2_ = (½, 0, 0) contribution is still very weak. The data were refined by integrating the *q*_1_ = (0, ½, ½) superstructure and the structure was solved in the triclinic system, space group 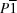, now with four crystallographically independent *o*-Me_2_TTF molecules (*A*–*D*) and two NO_3_^−^ anions (on atoms N1*A* and N1*B*), all in general positions. The lattice parameters (*a*, *b*′, *c*′) of the triclinic cell are connected to the high-symmetry lattice parameters of the monoclinic phase: *b*′ = *b* − *c* and *c*′ = *b* + *c* (Fig. 3 ▸ *b*). Each of the donor molecules generates, through inversion centres, stacks formed with only one independent *o*-Me_2_TTF molecule, *i.e.* *AA*, *BB*, *CC* and *DD* stacks, each of them dimerized as in the HT structure. As shown in Table 2 ▸, the degree of dimerization varies slightly from one stack to another, but each of them is smaller than the unique one of the HT structure. The presence of four different stacks does not modify appreciably the magnetic interactions within the chains (*J*) as no anomaly is observed at *T*_AO_ on the spin susceptibility. This point will allow us to use single-chain models in the description of the SP transition (see *Discussion* section[Sec sec4]).

Another interesting point is the potential evolution of the molecular charge within each of the four different molecules *A*–*D* of *o*-Me_2_TTF. Correlations between the intramolecular bond lengths and the charge have already been established for TTF or BEDT-TTF salts (Umland *et al.*, 1988 ▸; Guionneau *et al.*, 1997 ▸). A reliable molecular charge ρ is obtained with a formula which is written as ρ = *a* [(C_i_=C_i_)/(C_i_—S)] + *b*, where C_i_=C_i_ is the central C=C bond length and C_i_—S is the averaged C—S bond length involving the internal carbon atoms. These C_i_=C_i_ and C_i_—S distances are indeed the ones most affected by the oxidation of the TTF core (Katan, 1999 ▸). Note that more direct determination of ρ can be also obtained from intrinsic properties, *e.g.* the electronic structure, theoretically or experimentally (Oison *et al.*, 2003 ▸; García *et al.*, 2007 ▸). A linear fit based on the reported structural data for 13 compounds involving *o*-Me_2_TTF (see Table S1 in the supporting information) afforded *a* = 21.823 and *b* = −16.654 (*R* = 0.995). As shown in Table 3 ▸, application of this formula and normalization (so that the total charge of the organic molecules in the asymmetric unit is recovered) gives values close to 0.50 for the four molecules *A*–*D* of *o*-Me_2_TTF, excluding the formation of a sizeable charge-ordering process in this temperature range.

**Figure 7:**
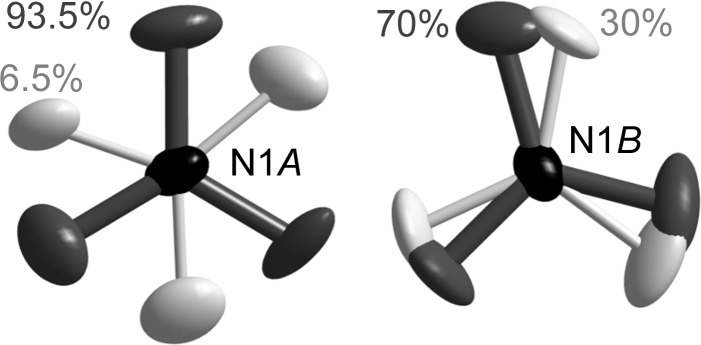
The disorder models used in the refinement of the NO_3_^−^ anion at 85 K in the *q*_1_ = (0, ½, ½) superstructure.

As shown in Fig. 3 ▸(*b*), the intermediate *q*_1_ = (0, ½, ½) superstructure is also associated with the presence of two different NO_3_^−^ anions, both of them being essentially ordered, as detailed in Fig. 7 ▸. The best refinements were obtained with one nitrate anion on atom N1*A* localized at 93.5% on one single position (the other position being rotated by 60°), and the other nitrate anion on atom N1*B* strongly agitated between two close positions. The main structural change related to the symmetry breaking is related to the anion ordering, which accompanies the ferroelastic phase transition from monoclinic to triclinic lattices. This structural phase transition is also accompanied by rotations of the long mol­ecular axis of the *o*-Me_2_TTF molecules in neighbouring stacks, shown as blue and red lines in Fig. 3 ▸(*b*).

#### 3.5.2. The *q*_1_ + *q*_2_ = (½, ½, ½) superstructure refinement   

**Figure 8:**
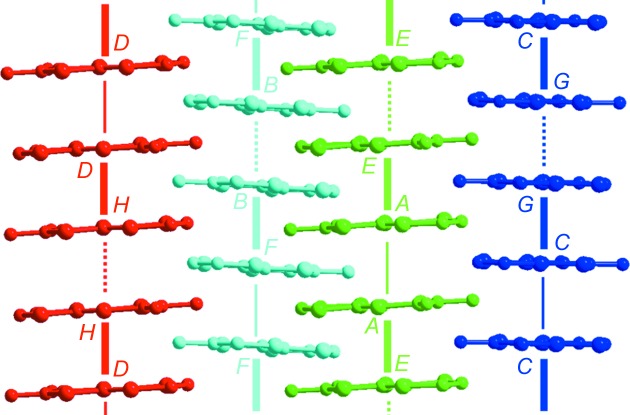
Views of the four tetramerized stacks with *AE*, *BF*, *CG* and *DH* dimers in the 20 K structure of (*o*-Me_2_TTF)_2_NO_3_.

The X-ray data collected at 20 K were refined within the *q*_1_ + *q*_2_ = (½, ½, ½) superstructure in the (*a*′, *b*′, *c*′) triclinic cell, also associated with a doubling of the stacking parameter (*a*′ = 2*a*). This triclinic cell, space group 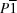, now contains eight crystallographically independent *o*-Me_2_TTF molecules (*A*–*H*) and four nitrate anions. As shown in Fig. 3 ▸(*c*), the anions are now fully ordered. The donor mol­ecules organize into four different stacks, formed of *AE*, *BF*, *CG* and *DH* dimers, respectively, and made of dissimilar molecules. The calculated overlap interaction energies within the four independent stacks are collected in Table 2 ▸ and the intermolecular plane-to-plane distances are given in Table S4 in the supporting information. It appears that:

(i) The stacks are now tetramerized (Fig. 8 ▸), with one strong overlapping intra-dimer interaction β_intra_ between dissimilar molecules (thick segments in Fig. 8 ▸ for the *AE*, *BF*, *CG* and *DH* pairs) and two weaker inter-dimer β_inter1_ and β_inter2_ interactions between the two types of similar molecules (thin and dotted segments in Fig. 8 ▸). By taking the average 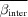 of β_inter1_ and β_inter2_, one can still define a degree of dimerization for the four stacks of the unit cell (see Table 2 ▸) which is slightly smaller than those of the *q*_1_ = (0, ½, ½) superstructure.

(ii) The β_inter1_ and β_inter2_ interactions are, respectively, stronger and weaker than the β_inter_ interaction found in the *q*_1_ = (0, ½, ½) superstructure. From the relative difference of the two inter-dimer overlap interactions, one can define a degree of tetramerization which is half of the degree of dimerization (Table 2 ▸).

(iii) There is a strong modulation of the charge of the *o*-Me_2_TTF molecules at 20 K from 0.2 to 0.8 (Table 3 ▸). This is a surprising result which should be confirmed by local spectroscopic measurements, because both inter-stack (inter-dimer) and intra-dimer charge transfers which spread the wave­function of *S* = ½ species should destabilize the spin-Peierls pairing mechanism (pairing of spin ½ into a magnetic singlet). This is in contrast with Fig. 5 ▸(*a*) showing the formation of well defined singlets below 96 K. However, the appearance of *q*_2_ is associated with the formation of antiphase domains and the long-range structural order may look incomplete.

## 4. Discussion   

### 4.1. Analysis of the anion-ordered structure   

Before discussing in more detail the electronic and magnetic properties of this salt, it is interesting to investigate how the ordering of the anions (which operates in the intermediate temperature regime) is associated with correlated movements of the donor molecules and particularly with the possible setting of weak C—H⋯O hydrogen bonds, as already observed in the halide salts (*o*-Me_2_TTF)_2_ *X* (*X* = Cl, Br, I; Foury-Leylekian *et al.*, 2011 ▸; Jankowski *et al.*, 2011 ▸; Reinheimer *et al.*, 2012 ▸). In the vicinity of the NO_3_^−^ anion, one finds: (i) sulfur atoms of the unsubstituted di­thiole ring (bearing H and not Me groups) of four different *o*-Me_2_TTF molecules; (ii) the corresponding ‘aromatic’ H atoms linked directly to *sp* ^2^ C atoms of this di­thiole ring; and (iii) the aliphatic H atoms of the methyl groups of a second set of four *o*-Me_2_TTF molecules. The shortest contacts are the S_TTF_⋯O ones (Table S3 in the supporting information), ranging from 2.86–3.05 Å at 250 K to 2.82–2.97 Å at 85 K and down to 2.80–3.04 Å at 20 K. These distances are indeed much shorter than the sum of the van der Waals radii (1.52 + 1.80 = 3.32 Å).

**Figure 9:**
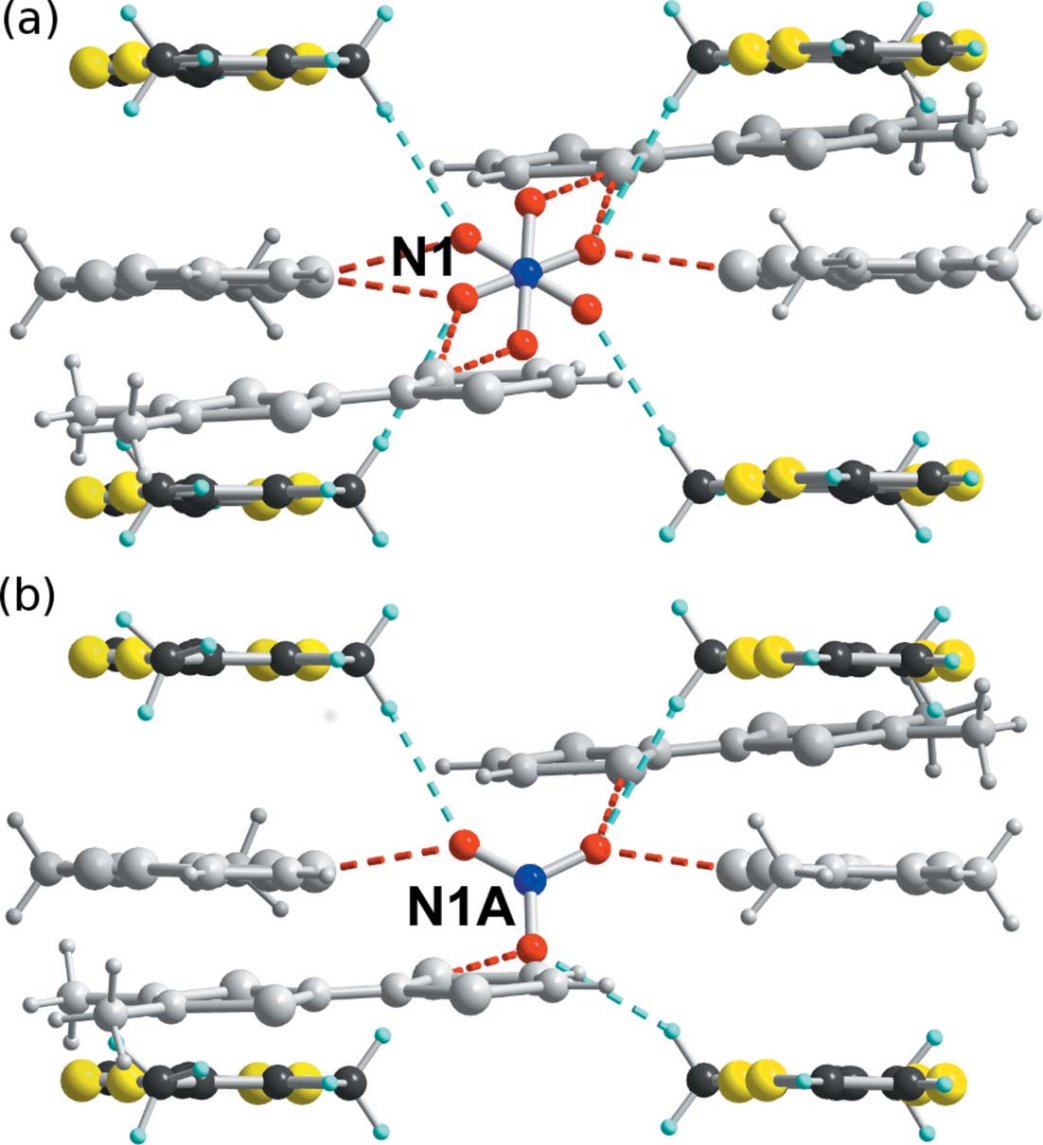
Details of the short S⋯O interactions (red dashed lines) and weak C—H⋯O hydrogen bonds (turquoise dashed lines) between the *o*-Me_2_TTF molecules and the NO_3_^−^ anion. (*a*) At 250 K. (*b*) In the intermediate *q*_1_ = (0, ½, ½) phase for the major NO_3_^−^ anion orientation on atom N1*A* (see Fig. 7 ▸). The *o*-Me_2_TTF molecules involved in S⋯O interactions and C_Me_—H⋯O hydrogen bonds are in grey and full colour, respectively.

Two types of hydrogen atom are available here, either aliphatic H atoms of the methyl groups or ‘aromatic’ H atoms directly linked to *sp* ^2^ C atoms of the TTF core. The shortest (C—)H⋯O interactions are found with the latter C*sp* ^2^—H hydrogen atoms, with short H⋯O distances between 2.40 and 2.45 Å but with poor directionality (C*sp* ^2^—H⋯O angles 115–118°; Fig. 9 ▸ *a*). In addition, this first ‘coordination’ sphere is complemented, at room temperature, with four other C_Me_—H⋯O interactions involving the methyl groups of four other neighbouring *o*-Me_2_TTF molecules, at 2.52 and 2.75 Å. The H⋯O distances are slightly longer but their directionality is much more pronounced (C_Me_—H⋯O angles 157–178°). Below the anion-ordering temperature, the inversion centre where the NO_3_^−^ anion is located at high temperature is lost. The badly oriented C*sp* ^2^—H⋯O interactions are elongated and weakened, while the four C_Me_—H⋯O contacts (identified at room temperature) transform into three well oriented contacts towards the three localized oxygen atoms of the two independent nitrate anions (Fig. 9 ▸ *b*). Note that stronger and more directional contacts are found around the nitrate on atom N2*A* than around the nitrate on atom N1*A* (Table S2 in the supporting information). Altogether, this analysis demonstrates that the ordering of the nitrate anions in the intermediate (0, ½, ½) phase is indeed associated with the anchoring of the nitrate anion through the setting of three directional C_Me_—H⋯O hydrogen bonds involving the methyl substituents.

These features are not modified in the low-temperature (½, ½, ½) phase with four different nitrate ions: the strongest interactions involve the nitrate ions on atoms N1*A* and N1*C*, and the weakest ones those on atoms N1*B* and N1*D*. It has recently been shown in systems exhibiting charge-ordered states (CO) that the anions have a tendency to move towards and/or to interact strongly with the most oxidized donor molecules (Pouget, 2012*b* ▸; Alemany *et al.*, 2012 ▸, 2014 ▸). Considering the distribution of partial charges within the eight independent molecules *A*–*H* of *o*-Me_2_TTF mentioned above (see Table 3 ▸), the question of the association of their charge differences with a concomitant modulation of the C—H⋯O interactions arises (Table S2 in the supporting information). However, this is not the case, because each of the four nitrate anions actually interacts with six different donor molecules among the eight *A*–*H*, with a distribution of partial charges for each of them.

Comparison of the projection views along **a** of the high-temperature (Fig. 3 ▸ *a*) and *q*_1_ = (0, ½, ½) (Fig. 3 ▸ *b*) structures shows that the anion ordering is also associated with a rotation of the long molecular axis of the *o*-Me_2_TTF stacks, responsible for the ferroelastic transition from monoclinic to triclinic and the cell doubling in the *bc* plane. In other words, the anion ordering also modifies the host cavity, to accommodate the two different NO_3_^−^ orientations. This original behaviour might be considered as a peculiarity of the planar NO_3_^−^ anion, by contrast with more globular tetrahedral anions such as ClO_4_^−^ or ReO_4_^−^.

### 4.2. Electronic and magnetic properties   

**Figure 10:**
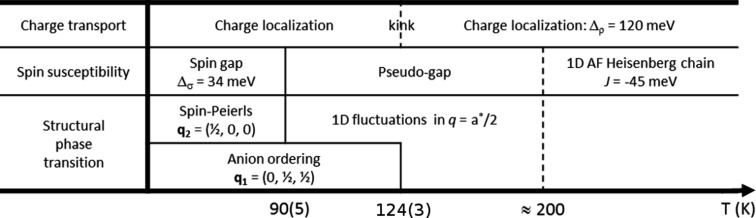
Combined structural and electronic properties of (*o*-Me_2_TTF)_2_NO_3_.

The experimental results in relation to the electronic and magnetic properties of (*o*-Me_2_TTF)_2_NO_3_ are summarized in Fig. 10 ▸.

The first surprising result in (*o*-Me_2_TTF)_2_NO_3_ is to obtain well decoupled anion-ordering (AO) and spin-Peierls (SP) transitions, since in the Fabre salts (TMTTF)_2_ *X* with *X* = ReO_4_, ClO_4_ and BF_4_, the unique *q* = (½, ½, ½) AO transition simultaneously achieves an SP-like ground state (Coulon *et al.*, 2015 ▸). Surprisingly, in the Fabre salt with *X* = NO_3_^−^, the spin-gap opening is shifted to a lower temperature than *T*_AO_ (Coulon *et al.*, 2015 ▸). However, in the absence of precise structural studies, the origin of this shift remains unclear in (TMTTF)_2_NO_3_. The situation is different in (*o*-Me_2_TTF)_2_NO_3_ presented here, because structural studies reveal an AO process in two steps. The upper AO critical temperature of (*o*-Me_2_TTF)_2_NO_3_, 124 (3) K, determined as the mean value of resistivity and diffraction results, is sizeably enhanced with respect to the *T*_AO_ values found for the Bechgaard and Fabre salts with the NO_3_^−^ anion (41 and 50 K, respectively; Pouget & Ravy, 1996 ▸). This is also the case for the SP critical temperature, 90 (5) K, which is much higher than that found in the Fabre salts with *X* = PF_6_ and AsF_6_ (Foury-Leylekian *et al.*, 2004 ▸; Pouget *et al.*, 2006 ▸) and in the (BCP-TTF)_2_ *X* series with the same anions (Liu *et al.*, 1993 ▸; Pouget & Ravy, 1996 ▸) (see Table 4 ▸).

Conductivity data over the whole temperature range below room temperature show that, with an activation energy of 0.12 eV (corresponding to about half the charge gap), the charge transport is more strongly activated than in the Fabre salts where an activated conductivity (*E*_act_ ≃ 0.03 eV) is detected only below *T*_ρ_ ≃ 200–250 K (Coulon *et al.*, 1982 ▸). The activated charge transport corresponds to a charge localization phenomenon on dimers due to strong electron–electron interactions (Giamarchi, 2004 ▸; Pouget, 2012*a* ▸, 2015 ▸). This finding is clearly related to the observation (Table 2 ▸) of an enhanced degree of dimerization of the one-dimensional stacks with respect to the Fabre salts (Pouget & Ravy, 1996 ▸). The conductivity data also exhibit a kink at the AO transition, which leads to a better conducting state below *T*_AO_. This observation can be easily understood by the simultaneous enhancement of the carrier mobility due to the suppression of the scattering potential of the disordered anions and a decrease in Δ_ρ_ associated with the slight reduction in the degree of dimerization below *T*_AO_ (see Table 2 ▸).

The AO transition is heralded by a narrow regime of three-dimensional critical structural fluctuations above *T*_AO_ (see Fig. 6 ▸ *c*), while the SP transition is preceded by a large thermal regime of quasi-one-dimensional structural fluctuations, which extend to *T*_fl_ ≃ 200 K. The three-dimensional anisotropy of these fluctuations indicates that the AO transition is achieved by a quasi-isotropic coupling between anions, as previously observed for the Bechgaard and Fabre salts (Pouget *et al.*, 1981 ▸, 1982 ▸), while the SP instability is driven by a one-dimensional electronic instability at *q* = a*/2, as previously observed in TMTTF (Pouget *et al.*, 1982 ▸) and BCP-TTF (Liu *et al.*, 1991 ▸, 1993 ▸) salts. The surprising result here is that these two kinds of instability appear to be thermally decoupled. In particular, the SP instability develops below *T*_fl_ ≃ 200 K in the temperature range where the anions are still disordered. It is useful to compare here the SP instability of (*o*-Me_2_TTF)_2_NO_3_ with the SP instability of other quarter-filled organic salts such as *d*_12_-(TMTTF)_2_PF_6_ (Pouget *et al.*, 2017 ▸) and (BCP-TTF)_2_AsF_6_ (Dumoulin *et al.*, 1996 ▸) which have been studied in detail. Table 4 ▸ summarizes the SP characteristics of these various salts.

The data of Table 4 ▸ show that the Δσ/*T*_SP_ ratio amounts to 4 in (*o*-Me_2_TTF)_2_NO_3_, as for (BCP-TTF)_2_AsF_6_. (Note that the mean-field ratio Δσ^MF^/*T*_SP_^MF^ for the SP transition of a spin ½ *AF* Heisenberg chain is 2.47; Orignac & Chitra, 2004 ▸). This ratio is smaller than that of 5.8 found for *d*_12_-(TMTTF)_2_PF_6_ (Pouget *et al.*, 2017 ▸). For this last compound, Table 4 ▸ shows that the SP gap is a small fraction of *J*, a value consistent with a small stack tetramerization of 3% (Kitou *et al.*, 2017 ▸) In this situation, the structural counterpart of the SP pairing along the stack direction induces a small modulation of the exchange integral *J*. This corresponds to a weak coupling situation, which is generally used in the literature to describe the SP transition (Bray *et al.*, 1983 ▸). The case of the other two salts where the SP gap is comparable with *J* is different. In particular, Δσ nearly amounts to *J* in (*o*-Me_2_TTF)_2_NO_3_, in agreement with the presence of a large stack tetramerization of 24% (see Table 2 ▸). This implies that the SP transition of this compound should be treated in the strong coupling limit. Generally, one obtains a first-order SP transition within this limit (Bray *et al.*, 1983 ▸). This is the case in the inorganic system VO_2_ and its alloys, where a strong dimerization of the Heisenberg chains is observed (Pouget *et al.*, 1974 ▸). However, the magnetic measurements (Fig. 5 ▸) show that the SP transition of (*o*-Me_2_TTF)_2_NO_3_ is a second-order transition. One possible explanation could be that organic materials incorporating anions in smooth cavities are particularly soft materials.

The SP transition in (*o*-Me_2_TTF)_2_NO_3_ is heralded by a sizeable regime of one-dimensional structural fluctuations below *T*_fl_ ≃ 200 K, which is manifest by the observation of diffuse lines at *q* = a*/2 in the X-ray patterns shown in Figs. 6 ▸(*b*) and 6 ▸(*c*). This diffuse scattering reflects the presence of local one-dimensional structural SP pairing in the stack direction (*i.e.* local tetramerization corresponding to a dimerization of the stack of dimers). This local pairing forms localized non-magnetic *S* = 0 singlets which induce a decrease in the spin susceptibility with respect to that of the uniform *S* = ½ *AF* Heisenberg chain. This deviation is clearly apparent in Fig. 5 ▸(*a*) below 200 K. This behaviour compares with that previously reported (Liu *et al.*, 1993 ▸) and theoretically calculated (Dumoulin *et al.*, 1996 ▸) for (BCP-TTF)_2_AsF_6_. Furthermore, this last calculation indicates that the mean-field SP temperature *T*_SP_^MF^ amounts to *T*_fl_. One-dimensional structural fluctuations form a pseudo-gap in the density of states of the magnetic excitations, which transforms into a real spin gap at *T*_SP_ (the three-dimensional SP transition) in the presence of inter-chain coupling. This scenario has recently been confirmed experimentally in *d*_12_-(TMTTF)_2_PF_6_ (Pouget *et al.*, 2017 ▸). At the mean-field SP temperature *T*_SP_^MF^, and using the mean-field ratio of 2.7, one obtains Δσ^MF^ ≃ 42.6 meV between *T*_fl_ and *T*_SP_. Δσ^MF^ is only slightly larger than Δσ ≃ 33.6 meV. This means that the reduction in spin gap due to quantum fluctuation is small, and thus that the SP transition of (*o*-Me_2_TTF)_2_NO_3_ occurs in the adiabatic (classical) regime. The same regime is found for (BCP-TTF)_2_AsF_6_ (Pouget, 2012*a* ▸). In contrast, the SP transition of *d*_12_-(TMTTF)_2_PF_6_ occurs just at the boundary with the anti-adiabatic (quantum) regime (Pouget *et al.*, 2017 ▸).

## 5. Conclusions   

We have unravelled here an original cation radical salt of the unsymmetrically substituted *o*-Me_2_TTF donor molecule. At variance with its halide salts (*X* = Cl^−^, Br^−^, I^−^) which exhibit uniform stacks with a three-quarter filled one-dimensional band structure and associated metallic conductivity, (*o*-Me_2_TTF)_2_NO_3_ forms dimerized stacks with a chessboard organization, but with the non-centrosymmetric nitrate anion disordered on an inversion centre. The combination of transport, magnetic and structural data evidences two successive transitions, an anion-ordering (AO) process associated with a *q*_1_ = (0, ½, ½) superstructure, and a spin-Peierls (SP) transition associated with a large stack tetramerization with a *q*_2_ = (½, 0, 0) superstructure. The surprising result here is that these two kinds of instability appear to be thermally decoupled, at variance with other TMTTF or TMTSF salts with non-centrosymmetric counter-ions. Indeed, the SP instability develops below *T*_fl_ ≃ 200 K in a temperature range where the anions are still disordered. Despite a large stack tetramerization (24%), the SP transition of (*o*-Me_2_TTF)_2_NO_3_ is still of second-order nature, a behaviour which finds its origin in the softness of these organic lattices, illustrated here by the adaptation of the weak C_Me_—H⋯O hydrogen-bond network to the anion-ordering process.

## Supplementary Material

Crystal structure: contains datablock(s) oDMTTF2NO3_250K, oDMTTF2NO3_085K, oDMTTF2NO3_020K. DOI: 10.1107/S2052252518004967/lc5098sup1.cif

Structure factors: contains datablock(s) oDMTTF2NO3_250K. DOI: 10.1107/S2052252518004967/lc5098oDMTTF2NO3_250Ksup2.hkl

Structure factors: contains datablock(s) oDMTTF2NO3_085K. DOI: 10.1107/S2052252518004967/lc5098oDMTTF2NO3_085Ksup3.hkl

Structure factors: contains datablock(s) oDMTTF2NO3_020K. DOI: 10.1107/S2052252518004967/lc5098oDMTTF2NO3_020Ksup4.hkl

Tables S1-S4. DOI: 10.1107/S2052252518004967/lc5098sup5.pdf

CCDC references: 1562992, 1562993, 1562994

## Figures and Tables

**Table 1:** Crystallographic data for (*o*-Me_2_TTF)_2_NO_3_ at different temperatures

Temperature	250 K	85 K	20 K
Formula	C_16_H_16_NO_3_S_8_	C_16_H_16_NO_3_S_8_	C_16_H_16_NO_3_S_8_
*M*_r_	526.78	526.78	526.78
Crystal colour	Black	Black	Black
Crystal size (mm)	0.202 × 0.153 × 0.03	0.202 × 0.153 × 0.03	0.308 × 0.145 × 0.069
Crystal system	Monoclinic	Triclinic	Triclinic
Space group	*P*2_1_/*c*	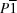	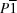
Cell	(*a*, *b*, *c*)	(*a*, *b*′, *c*′)	(*a*′, *b*′, *c*′)
*T* (K)	250.0 (1)	85.0 (2)	20 (2)
*a* or *a*′ (Å)	7.04980 (10)	6.9201 (2)	13.7401 (5)
*b* or *b*′ (Å)	12.1123 (2)	17.5521 (3)	17.4938 (6)
*c* or *c*′ (Å)	12.7923 (2)	17.5806 (3)	17.5169 (5)
α (°)	90.00	86.6840 (10)	86.488 (2)
β (°)	102.710 (2)	80.849 (2)	80.917 (3)
γ (°)	90.00	80.535 (2)	80.445 (3)
*V* (Å^3^)	1065.56 (3)	2078.40 (8)	4097.3 (2)
*Z*	2	4	8
*D*_calc_ (g cm^−3^)	1.642	1.683	1.708
λ (Å)	1.54184 (Cu *K*α)	1.54184 (Cu *K*α)	0.71073 (Mo *K*α)
μ (mm^−1^)	0.7938	0.814	0.892
Total No. of reflections	16535	31944	38892
Absorption correction	Multi-scan	Analytical	Analytical
*T*_max_, *T*_min_	1.0, 0.435	0.792, 0.333	0.946, 0.816
No. of unique reflections	2108	8189	17851
*R*_int_	0.0370	0.0395	0.0434
No. of unique reflections [*I* > 2σ(*I*)]	1998	7493	10365
No. of refined parameters	145	569	1025
*R*_1_ [*I* > 2σ(*I*)]	0.0237	0.0417	0.0479
*wR*_2_ (all data)	0.0665	0.1095	0.1517
Goodness-of-fit	1.071	1.186	1.032
Δρ_max_, Δρ_min_ (e^−^Å^−3^)	0.311, −0.198	0.58, −0.48	0.68, −0.89

*R*_1_ = Σ||*F*_o_| − |*F*_c_||/Σ|*F*_o_|; *wR*_2_ = [Σ*w*(*F*_o_^2^ − *F*_c_^2^)^2^/Σ*wF*_o_^4^]^1/2^.

**Table 2:** Calculated overlap interaction energies within the stacks in (*o*-Me_2_TTF)_2_NO_3_ in the different phases In the low-temperature (½, ½, ½) superstructure, each dimer indicated in parentheses is made of dissimilar molecules.

Structure	Stack motif	|β_intra_| (eV)	|β_inter_| (eV)	|β_inter2_| (eV)	Dimerization degree† ‡	Tetramerization degree§
Room temperature		0.724	0.388		0.60†	
						
(0, ½, ½)	*AA*	0.793	0.443		0.57†	
	*CC*	0.701	0.435		0.47†	
	*DD*	0.693	0.437		0.45†	
	*BB*	0.690	0.421		0.48†	
						
(½, ½, ½)	*B*(*BF*)*F*	0.823	0.538 (*FF*)	0.405 (*BB*)	0.54‡	0.28§
	*A*(*AE*)*E*	0.699	0.507 (*EE*)	0.400 (*AA*)	0.43‡	0.24§
	*D*(*DH*)*H*	0.694	0.477 (*DD*)	0.385 (*HH*)	0.47‡	0.21§
	*C*(*CG*)*G*	0.689	0.499 (*CC*)	0.391 (*GG*)	0.43‡	0.24§

† Defined as 2(β_intra_ − β_inter_)/(β_intra_ + β_inter_).

‡ Defined as in † but with 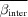 = (β_inter1_ + β_inter2_)/2 replacing β_inter_

§ Defined as (β_inter1_ − β_inter2_)/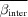.

**Table 3:** Calculated charges of the *o*-Me_2_TTF molecules at the three different temperatures, based on the application of the formula ρ = 21.823 × [(C_i_=C_i_)/(C_i_—S)] − 16.654

*T* (K)	Molecule	C_i_=C_i_ (Å)	Averaged C_i_—S (Å)†	Calculated charge (ρ_calc_)†	Normalized calculated charge (ρ_norm_)
250	*A*	1.364 (2)	1.735 (2)	0.50 (4)	0.5
					
85	*A*	1.368 (4)	1.737 (2)	0.54 (7)	0.52
	*B*	1.369 (4)	1.737 (2)	0.54 (7)	0.53
	*C*	1.362 (4)	1.740 (2)	0.43 (7)	0.42
	*D*	1.371 (4)	1.738 (2)	0.56 (7)	0.54
	Sum			2.07	2.00
					
20	*A*	1.344 (7)	1.740 (3)	0.20 (12)	0.18
	*E*	1.373 (8)	1.724 (3)	0.73 (12)	0.68
	*B*	1.373 (7)	1.734 (3)	0.63 (12)	0.58
	*F*	1.386 (7)	1.724 (3)	0.89 (12)	0.83
	*C*	1.363 (7)	1.734 (3)	0.50 (12)	0.47
	*G*	1.369 (7)	1.737 (3)	0.55 (12)	0.51
	*D*	1.357 (7)	1.733 (3)	0.42 (12)	0.40
	*H*	1.356 (8)	1.737 (3)	0.38 (12)	0.35
	Sum			4.293	4.00

† S.u.’s on the averaged C—S distances and on the calculated charges were evaluated using error propagation rules.

**Table 4:** Characteristics of various SP systems

Salt	Δσ (meV)	Stack tetramerization	*T*_SP_ (K)	*T*_fl_ ≃ *T*_SP_^MF^ (K)	|*J*| (meV)	|*J*|/Δσ
(*o*-Me_2_TTF)_2_NO_3_	33.6	0.24	90	200	44.8	1.3
*d*_12_-(TMTTF)_2_PF_6_	6.5	0.03	13.1	40	39	6
(BCP-TTF)_2_AsF_6_	11.5	Not defined	32.5	120	23.3	2
