# Supplementary material for: Decoupling anion-ordering and spin-Peierls transitions in a strongly one-dimensional organic conductor with a chessboard structure, (o-Me2TTF)2NO3
Source: IUCrJ. 2018 Apr 27;5(Pt 3):361–72. doi: 10.1107/S2052252518004967 (PMC5929382; doi:10.1107/S2052252518004967)
Supplement: Supplementary file 5 [file m-05-00361-sup5.pdf]

IUCrJ

Volume 5 (2018)

Supporting information for article:

Decoupling anion ordering and Spin-Peierls transitions in a strongly one-dimensional organic conductor with chessboard structure, (*o*-Me2TTF)2NO3

Olivier Jeannin, Eric W. Reinheimer, Pascale Foury-Leylekian, Jean-Paul Pouget, Pascale Auban-Senzier, Elzbieta Trzop, Eric Collet and Marc Fourmigué

Table S1. Structural characteristics of reported *o*-Me_2TTF salts, used to establish a formula correlating them with the charge (see text).

Compound	C_i=C_i	HC=CH	MeC=CMe	C_i–S	Charge	CSD code
o-DMTTF	1.340	1.308	1.338	1.768/1.765<br>1.753/1.761	0	NUNPOB
o-DMTTF/<br>*p*-Ph(NO_2)$_2$	1.342	1.315	1.335	1.761/1.758<br>1.750/1.751	0	JOKJEA
(*o*-DMTTF)$_2$/1,2,4,5-tetracyanobenzene ($P_{\bar{1}}$)	1.342	1.317	1.334	1.760/1.756<br>1.754/1.751	0	MUQKOZ
(*o*-DMTTF)$_2$/1,2,4,5-tetracyanobenzene ($P2_1/n$)	1.337	1.312	1.325	1.749/1.750<br>1.748/1.743	0	TOBSOU
o-DMTTF / 9-(5-Nitrofuran-2-ylidene)-2,4,5,7-tetranitrofluorene	1.345	1.333	1.387	1.762/1.778<br>1.754/1.767	0	VODGAW
(*o*-DMTTF)$_2$/Br	1.365	1.328	1.342	1.741<br>1.734	+0.5	NOHWUD
(*o*-DMTTF)$_2$/Cl	1.363	1.320	1.338	1.743<br>1.731	+0.5	NOHXAK
(*o*-DMTTF)$_2$/I	1.366	1.333	1.350	1.742<br>1.732	+0.5	NOHXEO
o-DMTTF/I_3	1.400	1.339	1.354	1.722/1.729<br>1.716/1.720	+1	QUJDEF
(*o*-DMTTF)$_2$/Re_2Cl_8	1.374<br>1.400	1.338<br>1.341	1.357<br>1.337	1.711/1.718<br>1.723/1.712<br>1.713/1.726<br>1.702/1.723	+1	SOJCAW
o-DMTTF/BF_4	1.386	1.336	1.355	1.725 / 1.723<br>1.720 / 1.718	+1	HOJQAZ
o-DMTTF/ReO_4	1.391	1.312	1.356	1.717<br>1.716	+1	WANJOK
(*o*-DMTTF)$_2$/W_6O_{19}	1.390	1.332	1.337	1.716/1.721<br>1.707/1.718	+1	KETNUU

Table S2 Structural characteristics of the weak C–H•••O hydrogen bonds between the sp2 and sp3 (methyl) hydrogen atoms of *o*-Me_2TTF and the oxygen atoms of the NO_3^- anions, at different temperatures (see also Figure 11 in manuscript).

T (K)	H atom's nature	N atom	Interacting atoms		H symmetry operation	(C–)H•••O distance (Å)	C–H•••O angle (°)
250 K	sp2 (×2)	N1	O1	H2	1-x, -0.5+y, 1.5-z	2.400(5)	117.7(2)
	sp2 (×2)	N1	O3	H3	1-x, -y, 1-z	2.446(10)	115.2(2)
	**sp3 (×2)**	N1	O2	H7A	x, 0.5-y, 0.5+z	2.524(9)	**178.3(2)**
	**sp3 (×2)**	N1	O1	H8C	x, -1+y, z	2.748(7)	**157.0(2)**
85 K	sp2	N1A	O2A	H3B	1-x, -y, 1-z	2.453(3)	112.6(2)
	sp2	N1A	O3A	H3C	x, y, 1+z	2.483(3)	110.2(2)
	sp2	N1A	O1A	H3D	x, y, 1+z	2.464(3)	115.2(2)
	sp2	N1A	O3A	H3A	1-x, 1-y, 1-z	2.633(3)	141.7(2)
	**sp3**	N1A	O3A	H8CC	1-x, 1-y, 1-z	2.549(4)	**172.7(2)**
	**sp3**	N1A	O2A	H8BA	1+x, y, z	2.777(4)	**159.7(2)**
	**sp3**	N1A	O1A	H8BC	x, y, z	2.758(4)	**152.1(2)**
	sp2	N2A	O3C	H2D	1-x, -y, -z	2.439(13)	119.0(3)
	sp2	N2A	O2C	H2A	x, y, z	2.516(9)	109.0(3)
	sp2	N2A	O1C	H2C	x, y, z	2.480(8)	113.8(3)
	sp2	N2A	O2C	H2B	1-x, -y, 1-z	2.827(14)	146.3(3)
	**sp3**	N2A	O2C	H7AA	1-x, 1-y, 1-z	2.502(14)	171.3(3)
	**sp3**	N2A	O3C	H7DC	1+x, y, z	2.555(13)	153.0(3)
	**sp3**	N2A	O1C	H7DA	x, y, z	2.673(16)	157.4(3)
20 K	sp2	N1A	O3A	H3B	x, y, z	2.423(4)	110.6(3)
	sp2	N1A	O2A	H3C	x, 1+y, z	2.504(4)	120.4(3)
	sp2	N1A	O2A	H3H	x, y, z	2.717(4)	106.4(3)
	sp2	N1A	O1A	H3E	x, y, 1+z	2.423(4)	116.9(3)
	**sp3**	N1A	O2A	H8CC	-x, 1-y, 2-z	2.463(6)	**149.7(3)**
	**sp3**	N1A	O3A	H8FA	x, y, z	2.541(5)	**172.0(4)**
	**sp3**	N1A	O1A	H8GC	x, y, 1+z	2.508(5)	**166.4(3)**
	sp2	N1C	O2C	H3D	x, y, z	2.726(4)	105.4(3)
	sp2	N1C	O1C	H3A	x, y, -1+z	2.469(4)	114.3(3)
	sp2	N1C	O2C	H3G	x, -1+y, z	2.499(4)	119.9(3)
	sp2	N1C	O3C	H3F	x, y, z	2.437(4)	109.9(3)
	**sp3**	N1C	O2C	H8GA	1-x, 1-y, -z	2.429(6)	**152.4(3)**
	**sp3**	N1C	O3C	H8BC	x, y, z	2.518(5)	**170.0(3)**
	**sp3**	N1C	O1C	H8CA	x, y, -1+z	2.583(5)	**156.9(3)**
	sp2	N1B	O1B	H2A	x, y, z	2.433(4)	110.7(3)
	sp2	N1B	O2B	H2D	x, y, z	2.460(3)	110.6(3)
	sp2	N1B	O1B	H2B	x, y, z	2.613(4)	142.8(2)
	sp2	N1B	O3B	H2C	x, y, z	2.448(4)	115.0(3)
	**sp3**	N1B	O3B	H7HC	x, -1+y, z	2.675(5)	**152.6(3)**
	**sp3**	N1B	O1B	H7EA	x, y, 1+z	2.540(5)	**169.3(3)**
	**sp3**	N1B	O2B	H7DC	1-x, -y, 1-z	2.717(5)	**164.6(3)**
	sp2	N1D	O1D	H2E	x, y, z	2.461(4)	109.2(3)
	sp2	N1D	O3D	H2H	x, y, z	2.459(3)	110.8(3)
	sp2	N1D	O2D	H2G	x, y, z	2.485(4)	113.8(3)
	sp2	N1D	O1D	H2F	x, y, z	2.555(4)	141.8(3)
	**sp3**	N1D	O1D	H7AC	x, y, -1+z	2.494(5)	173.2(3)
	**sp3**	N1D	O3D	H7HA	-x, 2-y, 1-z	2.730(5)	160.8(3)
	**sp3**	N1D	O2D	H7DA	x, 1+y, z	2.710(5)	154.6(3)

Table S3 S•••O intermolecular distances

Interacting atoms		O•••S dist. (Å)
T = 250K		
O1	S2 (1-x, -y, 1-z)	3.048(5)
	S1 (1-x, -0.5+y, 1.5-z)	3.025(5)
O2	S1 (1-x, -0.5+y, 1.5-z)	2.856(5)
O3	S2 (1-x, -y, 1-z)	2.964(6)
T = 85 K		
(N1A)–O1A	S2D (x, y, 1+z)	2.975(3)
(N1A)–O2A	S2A (1-x, 1-y, 1-z)	2.928(4)
	S2B (1-x, -y, 1-z)	2.884(3)
(N1A)–O3A	S2C (x, y, 1+z)	2.841(4)
(N1B)–O1C	S1C	2.960(12)
(N1B)–O2C	S1A	2.817(12)
(N1B)–O3C	S1B (1-x, -y, 1-z)	2.897(12)
	S1D (1-x, -y, -z)	3.132(13)
T = 20 K		
(N1A)–O1A	S2E (x, y, 1+z)	3.036(5)
(N1A)–O2A	S2H	2.849(5)
	S2C (x, 1+y, z)	3.273(5)
(N1A)–O3A	S2B	2.824(6)
(N1B)–O1B	S1A	2.846(6)
(N1B)–O2B	S1B	2.901(5)
	S1D	2.835(5)
(N1B)–O3B	S1C	2.967(5)
(N1C)–O1C	S2A (x, y, -1+z)	3.004(5)
(N1C)–O2C	S2G (x, -1+y, z)	3.229(5)
	S2D	2.839(5)
(N1C)–O3C	S2F	2.800(6)
(N1D)–O1D	S1E	2.840(6)
(N1D)–O2D	S1G	2.967(5)
(N1D)–O3D	S1F	2.877(5)
	S1H	2.870(5)

Table S4. Plane-to plane distances D (in Å) between *o*-Me_2TTF molecules in the 20K structure.

Interaction	Nature	β (eV)	D (Å)
DH	eclipsed	0.694	3.41
DD	bond-over-ring	0.477	3.41
HH	bond-over-ring	0.385	3.47
BF	eclipsed	0.823	3.45
FF	bond-over-ring	0.538	3.34
BB	bond-over-ring	0.405	3.41
AE	eclipsed	0.699	3.42
EE	bond-over-ring	0.507	3.42
AA	bond-over-ring	0.400	3.47
CG	eclipsed	0.689	3.42
CC	bond-over-ring	0.499	3.43
GG	bond-over-ring	0.391	3.46
